# Developing Community-Based Rehabilitation Programs for Musculoskeletal Diseases in Low-Income Areas of Mexico: The Community-Based Rehabilitation for Low-Income Communities Living With Rheumatic Diseases (CONCORD) Protocol

**DOI:** 10.2196/resprot.3604

**Published:** 2014-11-21

**Authors:** Adalberto Loyola Sánchez, Julie Richardson, Ingris Peláez-Ballestas, John N Lavis, Seanne Wilkins, Michael G Wilson, Jacqueline Rodríguez-Amado, José Alvarez-Nemegyei, Rebeca T Martínez-Villarreal, Dora J Onofre-Rodríguez, Raquel Benavides-Torres

**Affiliations:** ^1^School of Rehabilitation ScienceFaculty of Health SciencesMcMaster UniversityHamilton, ONCanada; ^2^Departamento de ReumatologíaHospital General de MéxicoMexico CityMexico; ^3^McMaster Health ForumMcMaster UniversityHamilton, ONCanada; ^4^Department of Clinical Epidemiology and BiostatisticsMcMaster UniversityHamilton, ONCanada; ^5^Centre for Health Economics and Policy AnalysisMcMaster UniversityHamilton, ONCanada; ^6^Department of Political ScienceMcMaster UniversityHamilton, ONCanada; ^7^Department of Global Health and PopulationHarvard School of Public HealthHarvard UniversityBoston, MAUnited States; ^8^Departamento de ReumatologiaHospital UniversitarioUniversidad Autonoma de Nuevo LeonMonterreyMexico; ^9^Unidad de InvestigaciónHospital Regional de Alta Especialidad de la Península de YucatánMéridaMexico; ^10^Centro Universitario de SaludFacultad de MedicinaUniversidad Autonoma de Nuevo LeonMonterreyMexico; ^11^Centro de Investigación y Desarrollo en Ciencias de la SaludFacultad de EnfermeriaUniversidad Autonoma de Nuevo LeonMonterreyMexico

**Keywords:** rehabilitation, community-based participatory research, social change, musculoskeletal diseases, Mexico

## Abstract

**Background:**

The negative impact of musculoskeletal diseases on the physical function and quality of life of people living in developing countries is considerable. This disabling effect is even more marked in low-socioeconomic communities within developing countries. In Mexico, there is a need to create community-based rehabilitation programs for people living with musculoskeletal diseases in low-socioeconomic areas. These programs should be directed to prevent and decrease disability, accommodating the specific local culture of communities.

**Objective:**

The objective of this paper is to describe a research protocol designed to develop, implement, and evaluate culturally sensitive community-based rehabilitation programs aiming to decrease disability of people living with musculoskeletal diseases in two low-income Mexican communities.

**Methods:**

A community-based participatory research approach is proposed, including multi and transdisciplinary efforts among the community, medical anthropology, and the health sciences. The project is structured in 4 main stages: (1) situation analysis, (2) program development, (3) program implementation, and (4) program evaluation. Each stage includes the use of quantitative and qualitative methods (mixed method program).

**Results:**

So far, we obtained resources from a Mexican federal agency and completed stage one of the project at Chankom, Yucatán. We are currently receiving funding from an international agency to complete stage two at this same location. We expect that the project at Chankom will be concluded by December of 2017. On the other hand, we just started the execution of stage one at Nuevo León with funding from a Mexican federal agency. We expect to conclude the project at this site by September of 2018.

**Conclusions:**

Using a community-based participatory research approach and a mixed method program could result in the creation of culturally sensitive community-based rehabilitation programs that promote community development and decrease the disabling effects of musculoskeletal diseases within two low-income Mexican communities.

## Introduction

### Musculoskeletal Diseases and Disability

Musculoskeletal diseases are highly prevalent in communities of many developed and developing countries, resulting in important health problems for individuals and society [1-4]. Many epidemiological studies performed in developed countries have found high levels of disability and work absenteeism among people who suffer musculoskeletal disorders [5-8]. Furthermore, disability produced by musculoskeletal pain has a negative impact on the social and emotional well-being of people [9], especially in the older adult population [10].

The negative impact of musculoskeletal diseases on the physical function and quality of life of people is more marked within developing countries [11]. This could be linked to observations that increased disability is associated with lower socioeconomic levels [12,13]. A large epidemiological study conducted in Mexico reported that the prevalence of musculoskeletal pain was 26%, which was associated with 13% of physical disability [14]. This study also found significant differences in the regional prevalence of musculoskeletal pain and its causes across the country, implying the influence of different cultural, socioeconomic, and demographic factors within each geographical location [14].

In the Mexican northern state of Nuevo León, the prevalence of osteoarthritis is 17% [15], while the estimated national prevalence is 10.5% [14]. This shows that osteoarthritis is an important health problem for this region. In the southern state of Yucatán, the existence of chronic musculoskeletal diseases, such as osteoarthritis, back pain, and rheumatoid arthritis, produces a 6% prevalence of disability negatively affecting the life of the people living in this region [16,17]. As a result, there is a growing interest in designing community-level interventions directed to decrease the musculoskeletal-related disability within these Mexican regions.

### Rehabilitation Interventions

Specifically, the health professionals of the University Health Center of Nuevo León (UHC-Nuevo León) have a particular interest in addressing the health problems posed by musculoskeletal diseases in their community. The UHC-Nuevo León is a primary health care program run by the Autonomous University of Nuevo León that provides health services to a large community of low socioeconomic level. On the other hand, the Latin American Group for the Study of Rheumatic Conditions in Indigenous People (Grupo Latinoamericano para el Estudio de Enfermedades Reumaticas en Poblaciones de Origen, GLADERPO) is interested in creating interventions for decreasing the disabling effects of musculoskeletal diseases in a municipality called Chankom, which is an underserved Mayan community located in the state of Yucatan. Consequently, these two groups are looking to design rehabilitation interventions aimed to address the musculoskeletal-related disability within their communities of interest.

Rehabilitation is defined as an “enabling” process aimed at reversing the “disabling” effects of a pathological condition [18] or a social situation [19]. This process involves efforts directed both at the persons and their environments, allowing them to get “back on track” with their lives and to achieve equal opportunities to participate in their desired social roles [20]. There is evidence that rehabilitation is effective at reducing the burden of disability, enhancing opportunities for disabled people. This results in an improvement of quality of life to the extent that the United Nations and the European Board of Physical and Rehabilitation Medicine consider “access to rehabilitation” as a human right [21,22].

Particularly, rehabilitation interventions have proven effective to decrease pain and improve physical function with people suffering from rheumatologic diseases [23]. Nevertheless, in Mexico only 1.7% of the people who suffer from musculoskeletal diseases receive rehabilitation [14]. Consequently, there is a need to develop community rehabilitation programs directed at decreasing the disabling effects of musculoskeletal diseases in both the community served by the UHC-Nuevo León (community-UHC-Nuevo León) and the Mayan community of Chankom.

### Community-Based Rehabilitation and Community-Based Participatory Research

The concept of community-based rehabilitation (CBR) has evolved over 30 years of community work, mostly in developing countries. CBR started as an approach of biomedical service and gradually progressed to a “human-rights” approach supporting community development [24]. Therefore, this approach is now defined as a community development strategy for the social inclusion of people with disabilities through the equalization of opportunities [25]. Due to its participatory focus it has been proposed that CBR is a “democratic tool for social change” [26].

Nevertheless, there have been some limitations in the application of the CBR approach worldwide, which include a lack of cultural sensitivity [24]. Cultural sensitivity refers to the ability to accommodate a specific culture [27], and successful community programs address this by including the knowledge, beliefs, and values of the target community [28]. Therefore, CBR programs should be culturally sensitive; in other words they need to be developed with primary consideration of the beliefs, perceptions, and values of the culture of the community where they will be implemented.

The concept of cultural sensitivity obtains significant relevance when dealing with very different communities, as in the case of the community-UHC-Nuevo León and Chankom. The 5 community health centers that form the community-UHC-Nuevo León provide care to 52 urban neighborhoods (approximately 140,000 persons). The entire population of this community speaks Spanish and belongs to a low to middle-low socioeconomic level. On the other hand, the community of Chankom Municipality has 4340 inhabitants spread across 11 small rural settlements or commissariats. The majority of Chankom’s population speaks Mayan and lives in very high levels of poverty. Given the sociocultural differences between these two communities, it is essential to adopt the concept of cultural sensitivity, and not to take a “one size fits all” approach for the development of the CBR programs.

Another important limitation of the CBR approach is the lack of formal research and scientific evaluation of its goals and processes [24,29-31]. Culturally sensitive CBR programs can be achieved through a “full and effective participation of the community” [25]. Consequently, participatory research strategies could represent a viable alternative to do research on CBR. There is one strategy, the community-based participatory research (CBPR), which has been proposed as an optimal method to develop culturally sensitive community-based health programs [32]. This strategy is part of the “participatory action” research that conceptualizes reality as formed by objective and subjective perspectives. Perspectives are historically constituted and reconstituted by human agency and social action, which implies a need to establish a dialectic relationship among different forms of knowledge production [33]. As a result, the CBPR approach involves the use of different quantitative and qualitative strategies to generate knowledge, which then can be used to address community needs [34,35].

CBPR is based on the following principles: (1) acknowledgment of the community as a unit of identity, (2) development of community strengths and resources, (3) promotion and facilitation of equitable and participatory partnerships with community members in all phases of research, (4) promotion of colearning and capacity building for all partnership members, (5) achievement of balance between knowledge generation and intervention for the mutual benefit of all partners, (6) focus on relevant problems for the community, (7) use of iterative and cyclical processes in all research, (8) involvement of all partners in the local and global dissemination of results, and (9) establishment of long-term commitment with partnership sustainability [32]. The application of these principles can result in knowledge that is owned by the community and is useful for the design, implementation, and evaluation of community interventions [32,35].

The use of CBPR strategies has resulted in increments of community capacity and positive effects on community health [36]. In Latin America, there is a long history of health-program development efforts through social participation, which have repeatedly failed to achieve all their goals [37]. Lessons learned from these experiences suggest that collaborative efforts established between communities and nongovernment institutions, such as universities, are an efficient way to solve immediate health issues, improve resource utilization, and raise social and political awareness [37]. In consequence, a CBPR strategy that includes alliances between community and academic institutions could be effective, producing structured social participation to solve disability related problems in the community-UHC-Nuevo León and the Municipality of Chankom.

### Main Intention and Objective

It has been stated that what really defines a social participation approach are the intentions and meanings given to the actions conducted by the people involved in it, and it is extremely important to be transparent about the intentions of using such a research strategy [38]. The main intention of this project is to organize and empower communities to develop a culturally sensitive CBR (csCBR) program in partnership with academics. This partnership will seek to form alliances with government and nongovernment institutions in order to ensure the success and continuation of the program. The csCBR program will aim to reduce the disabling effects of musculoskeletal diseases through supporting the efficient use of resources available in the communities and promoting micro and macro social changes.

The main objective of this protocol, which we named Community Based Rehabilitation for Low Income Communities Living With Rheumatic Diseases (CONCORD), is to develop, implement, and evaluate csCBR programs to decrease disability of people living with musculoskeletal diseases in the community-UHC-Nuevo León and the Municipality of Chankom. The hypothesis of this project is, “The execution of a CBPR strategy that permits a fusion of global and local knowledge will result in the creation of csCBR programs that will promote community development, thereby increasing social integration of disabled people with musculoskeletal diseases living in the communities of interest”.

### Theoretical Approach

The theoretical approach of this research project aligns with a social constructivist worldview, assuming that a successful CBR program can be developed through the construction of “new knowledge”. This new knowledge results from the “fusion of horizons” [39] between global knowledge (scientific/academic) and local knowledge (community beliefs and values). The new knowledge will be supported by community and academic members and will permit the definition of actions to facilitate its use for the benefit of the community. These actions will be structured as a complex intervention [40] in the form of a csCBR program and will involve collaborations with representatives of social and health policy institutions. In addition, we will use critical analytic approaches to disclose and resolve conflicts of power innate to every participatory action project.

## Methods

### Research Strategy and Methodology Overview

**Figure 1:**
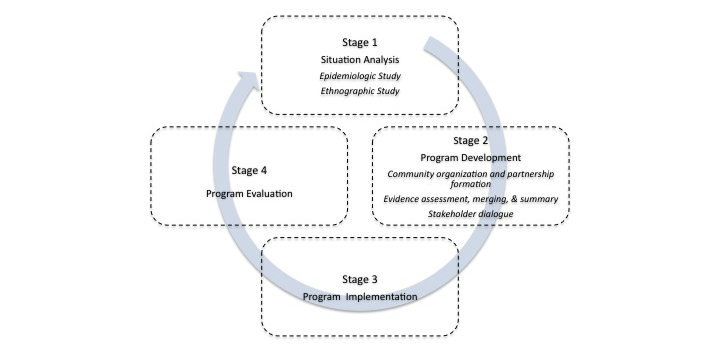
The “Community-Based Rehabilitation for Low Income Communities Living With Rheumatic Diseases (CONCORD)” protocol.

The CBPR strategy in this project will include a multi and transdisciplinary effort that involves a dialogic relationship between medical anthropology and some health sciences such as rheumatology, epidemiology, rehabilitation, nursing, and primary health care. Following the 2010 World Health Organization (WHO) guidelines for the development of CBR programs [41], this project is structured in four main stages (Figure 1 shows these stages). Methodologically, this project is conceived as a mixed method program, which involves the use of quantitative and qualitative methods in all its stages [42]. Following is a description of the main methodological elements that constitutes each of these stages. Differences on how each stage will be conducted in each of the communities of interest will also be noted.

### Stage One: Situation Analysis

#### Objectives

This stage will be undertaken over a 6-month period with the objectives of: (1) generating knowledge about the physical function problems produced by musculoskeletal diseases in the target communities; and (2) understanding the specific contexts in which these problems occur within each community. To achieve these objectives we will conduct an epidemiologic study in parallel with an ethnographic study.

#### Epidemiologic Study

This will be a “pure quantitative” study [42] with the specific objectives of: (1) assessing the prevalence and factors associated with musculoskeletal diseases development and progression in both communities of interest; and (2) evaluating the impact of musculoskeletal diseases on the physical function and health status of the people living in both communities. We expect to understand the impact of musculoskeletal diseases within the communities and to identify the presence of potentially modifiable factors to prevent or decrease disability.

This will be an observational, cross-sectional, survey-based study. Due to the different population sizes, in Chankom we will conduct a census of all adults (≥18 years old) living in the community; whereas in the community-UHC-Nuevo León we will obtain a multistage probabilistic sample of 1516 adults (considering a precision of 3%, a 95% confidence level, an estimated osteoarthritis prevalence of 20%, observed in Nuevo León State, and the sample size adjustment recommended for multistage sampling procedures) [43]. The primary sampling unit of the probabilistic sampling will be neighborhoods served by the UHC-Nuevo León. The secondary sampling unit will be blocks within selected neighborhoods, and the tertiary sampling unit will be households within selected blocks. All procedures will be performed using a random-start systematic proportional sampling procedure. In order to control for within-household homogeneity, we will only survey one person per household.

The survey procedure will be structured following the Community Oriented Program for the Control of Rheumatic Diseases (COPCORD) methodology [44]. The COPCORD is a screening strategy to detect rheumatologic disorders in the community and has proven to be effective when used in Mexico [45]. Briefly, the survey consists of a questionnaire designed to explore the presence of joint pain, stiffness, and inflammation along with factors associated with musculoskeletal diseases, physical activity, physical function, and health status.

Physical activity will be assessed using the well validated Mexican-Spanish version of the Rapid Assessment of Physical Activity questionnaire [46,47]. Health status will be evaluated through directly asking the participant, “How have you been with your illness?”, and physical function will be measured through the Health Assessment Questionnaire Disability Index (HAQ-DI). This questionnaire, which has shown good psychometric properties when applied to people with musculoskeletal chronic diseases [48], is available in Spanish [49] and has been validated within the Mexican population [50,51]. The survey will also include a socioeconomic assessment including education, income, home characteristics, and commodities.

Trained personnel will administer the survey to both communities in person. In the case of Chankom, a cross-cultural adaptation of the instrument to the Mayan language was conducted [52]. A trained general physician will assess all adults that reported any musculoskeletal symptoms at their homes using standardized criteria for the diagnosis of rheumatologic diseases. A specialist (rheumatologist or physiatrist) will confirm all cases identified with rheumatologic diseases.

The specialist will conduct a thorough medical assessment of all confirmed cases. This assessment will include radiographic evaluation, medical history, and physical examination with the objectives of evaluating the impact of disease on physical function and the presence of factors for functional decline and disease progression.

Physical function will be evaluated according to Glass’s tenses of “human functioning” [53]. These tenses are: (1) “enacted tense” or performance of meaningful activities within life context; (2) “hypothetical tense” or perceived capacity to do predefined activities; and (3) “experimental tense” or capability to do activities in standardized conditions. Performance of meaningful activities will be evaluated by self-report of main housework, work, and leisure activities, including an assessment of the concept “preclinical disability”. Preclinical disability refers to the state in which, in spite of no interruptions in the execution of regular activities, there is a modification of the way and/or the frequency in which these activities are performed [54]. Perceived capacity to do predefined activities will be assessed using the HAQ-DI, described above. Finally, capability to do activities in standardized conditions will be evaluated through the 6-minute walk test (6MWT) and the functional dexterity test (FDT). The 6MWT measures the distance an individual can walk during 6 minutes on a hard, flat surface [55], and has shown good test-retest reliability when used with people with musculoskeletal conditions such as osteoarthritis [56,57]. The FDT evaluates the ability to use the hand for “functional daily tasks that require 3-jaw chuck prehension between the fingers and the thumb” [58] and has shown good intra and interrater reliabilities and construct-validity in diverse pathologic conditions of the hand [59].

A member of the research team will perform periodic screenings to ensure the quality of the database. We will estimate descriptive statistics (central and dispersion estimates). In the case of the community-UHC-Nuevo León, we will also estimate 95% confidence intervals correcting for the three-stage sampling. We will use linear and logistic regression models to evaluate the factors associated with disease presentation and with impact on health and physical function utilizing specialized statistical software (STATA version 12).

#### Ethnographic Study

This “pure qualitative” study [42] will be conducted over 6 months, in parallel with the epidemiologic study, with the objective to produce an ethnographically informed report on the “explanatory models of illness” within the medical-anthropological “health systems” [60] of Chankom and the community-UHC-Nuevo León. Explanatory models of illness refer to the different narratives present on the causes, manifestations, trajectories, and treatments of disease, whereas the medical-anthropological health systems include the popular, traditional, and professional contexts in which health is conceived [61].

We will conduct a study from the perspective of ethnography [62]. This implies the conducting of fieldwork where anthropologists and other researchers-in-training will live in or close by the target communities. Given the high rates of violence registered in Nuevo León during the last few years, we will conduct preliminary in-depth interviews and focus groups with community leaders and health providers of the UHC-Nuevo León to ensure that it is safe for a researcher to live in this area, and in case it is not, to define alternative strategies to complete the planned fieldwork.

The fieldwork will include purposeful sampling of key persons, activities, social and familiar events, and documents. Key persons will include: (1) community members who have musculoskeletal diseases involving different body regions; (2) community leaders involved in community development activities; (3) representatives of health professionals involved in the care of people with musculoskeletal diseases in these communities; (4) representatives of health providers not officially recognized by a professional association (eg, bonesetters, masseurs, etc); (5) representatives of the local government; and (6) representatives of social development institutions (state and nongovernmental). These persons will be interviewed through informal and formal (in-depth interviews and focus groups) techniques.

We will perform participant and nonparticipant observations of individual’s activities (eg, occupation) and social, familial, cultural, and provincial events. These observations will be chosen according to their relevance to the musculoskeletal disability problematic within each community. Finally, we will obtain written documents that are relevant to understand the problem of musculoskeletal disability within each community (eg, local disability laws, social welfare rules, clinical practice guidelines, advertisements, etc).

All activities in Chankom will be conducted using Mayan translators who are fluent in Spanish and Mayan languages and are recognized by the community as members of their own. Access to each community will be negotiated with community leaders and local authorities. Data will be recorded by the use of field notes and audiotape recorders. We will aim to achieve thematic and/or theoretical saturation [63]. All data will be transformed to written electronic format and will be organized and managed using specialized qualitative data software (Hyperresearch, version 3.5.2).

Data will be analyzed and interpreted by the research team. The team will work on concept generation, typology development, and execution of comparative strategies. Constant reflection about team members’ emotions and prejudices that emerge while conducting the fieldwork will be executed. Data analysis and interpretation will be done through a continuous cycle of analysis-interpretation-reflection. The analysis-interpretation phase will feed into the data acquisition phase; hence they will occur simultaneously. An iterative analytic-interpretative process will be use in which theoretical ideas will be used to make sense of data and the data will be used to change theoretical ideas [64]. All analytic, interpretative, and methodological decisions will be carefully registered as memoranda within an audit trail book.

Completing this ethnography will help us understand the disability problematic caused by musculoskeletal disorders in the communities of interest. This study will allow the identification of barriers and facilitators for the optimal function of the population who suffers from musculoskeletal diseases in Chankom and the community-UHC-Nuevo León. Understanding the local culture and the native perspective on the causes, management, impact, and prognosis of musculoskeletal diseases will help us define better the problematic related to musculoskeletal diseases within the communities. In addition, knowing the communities’ local, regional, and national social structures along with their functional dynamics will orient us on how to proceed during the following stages of the project.

### Stage Two: Program Development

#### Objectives

This stage will take 12 months to complete and has the following objectives: (1) to organize the communities and form a partnership among these and members of academia under the principles of equity and mutual respect; (2) to define the priority problems related to the disabling effects of musculoskeletal diseases, and to identify possible solutions to these problems; and (3) to define the components of the csCBR program along with the necessary actions to implement them, assuring the necessary resources to execute them. This stage will follow a “qualitative dominant” methods perspective [42] being composed of three main and sequential activities: (1) community organization and partnership formation; (2) evidence assessment, merging, and summary; and (3) stakeholder deliberation. In addition, an anthropologist will conduct ethnographic work including nonparticipant observations and in-depth interviews on all these activities in order to produce a reflective-critical analysis from a medical anthropology perspective.

#### Community Organization and Partnership Formation

We will present the information gathered during the initial stage of this project to the community through the organization of community meetings at different strategic locations. During these meetings we will form 2 types of committees labeled as “first-level” or “second-level” committees. In Chankom, we will hold 11 meetings, one at each commissariat, and in the community-UHC-Nuevo León we will conduct 5 meetings, one at each of the health care units that form this center. These information meetings have the goal of creating awareness about the disabling effects of the musculoskeletal conditions explored within these communities. By the end of each meeting we will ask the community to choose 4 persons to constitute a first-level committee. A person from each of these first-level committees will participate in the second-level committee. There will be only one second-level committee, which includes representatives of all the strategic locations within our target communities (11 in Chankom and 5 in the community-UHC-Nuevo León).

The second-level committee of each community will be legally constituted as a “civil association”. This will be important for allocating and requesting financial resources, because in Mexico most government and nongovernment institutions can only serve organizations of this kind. The second-level committee will directly interact and work with representatives of the academic institutions involved in this project. During the first meeting of all committees, the members will define their roles as well as the rules for collaboration in relation to the processes of communication, decision making, and conflict resolution. We will use a nominal group technique, which is a group decision-making method, based on procedures for ideas’ exposition, discussion, and ranking that allows everyone’s opinion to be taken into account, reaching the best possible solution that is constituted by a mixture of all group members’ ideas [65].

The second-level committee and the academics will be in charge of all methodological and administrative decisions for the project, as they will take on the role of the principal investigator. All decisions taken within this partnership between communities and academia, from now on referred to as “the partnership”, will be the result of an ongoing analytic-interpretive-consensus process. In addition, the information and decisions generated within the partnership will be disseminated to the community via the first-level committees. In the same token, the community will be able to communicate with the second-level committee and academics through the first-level committees.

#### Evidence Assessment, Merging, and Summary

The first task for the partnership and the first-level committees will be to define the priority problems within their communities. The groups will use the knowledge generated during stage one of this project and the elements described by the WHO CBR matrix [41]. Priority problems refer to those issues that need to be urgently solved in order to decrease the disabling effects of musculoskeletal diseases at Chankom, and the community-UHC-Nuevo León. These issues will be organized and structured according to their main content in: (1) health, (2) education, (3) livelihood, (4) social, and (5) empowerment problems. The prioritization of problems will be based on their impact on the community’s health and physical function. We will then think about possible solutions using both, the communities’ social and cultural knowledge (local evidence) and the knowledge generated within the “scientific-academic” world (global evidence). These ideas will redefine community priority problems based on the cost, benefit, and efforts required to implement them.

The global evidence assessment will largely be the responsibility of the academic partners. This will be accomplished by combining the methodology for “overview of reviews” proposed by the Cochrane Collaboration [66] and the “evidence assessment” approach proposed by the “Grading of Recommendations Assessment, Development and Evaluation” group [67]. Once processed, this evidence will be formatted into a fourth grade level of comprehension, so every member of the partnership and committees can understand it. Following this, the partnership will merge both local and global evidences in order to construct a plan to solve the disabling problems posed by musculoskeletal diseases in the communities. Therefore, it is expected that this plan will be both solid, in relation to its scientific foundation, and sensitive to the cultural and social realities of each of the target communities.

Priority problems and the plan to attend them will be defined and written as an evidence brief (ie, a document that summarizes how the available evidence pertains to a pressing problem, select options for addressing the problem, and key implementation considerations). This evidence brief will be structured following the ideas developed by the McMaster Health Forum [68,69], along with ideas from the “scenario planning” strategy for organization planning [70]. These briefs will include: (1) a clear description of each problem including its context; (2) a description of possible individual, community, programmatic, and systemic solutions to address each problem through the use of different scenarios; (3) a description of expected outcomes (benefits, costs, and harms) for each scenario; (4) a simple description of the grade of uncertainty behind the expected outcomes of each scenario; (5) a description of possible barriers for the implementation of each possible solution; and (6) a clear description about the sources from which the information of the possible solutions and scenarios came.

#### Stakeholder Dialogue

The components of the csCBR program will be defined using the principles of the Communicative Action Theory, which assumes that communication aimed at reaching agreement is the base from which to coordinate the activities of social change [71]. Consequently, we will create a space for communication or forum to convene a stakeholder dialogue to support action for improving health outcomes through collective problem solving by different key decision makers. The stakeholder dialogue will be conducted based on the methods developed by the McMaster Health Forum [72]. In addition, we will attempt to achieve an “unforced consensus” [33], a goal not usually targeted by these kinds of forums. This consensus will be fundamental to assure the execution and sustainability of the csCBR program.

Key decision makers are defined as those knowledge users who are able to influence the decision-making processes of their respective areas. The partnership will identify key decision makers using the information gathered during the previous stage and substages of the project. We anticipate that identified key decision makers will represent at least one of the following areas: (1) traditional medicine, (2) professional health care, (3) government and nongovernment social welfare, and (4) health policy. During this part of the project we will intend to form an alliance with these key decision makers in order to create commitments that will ensure human and material resources for the execution of the csCBR program, independently from resources of this research project. We will recognize these key decision makers as “powerful allies”, based on the privileged position of power they held within their respective areas. Potential powerful allies will be invited to participate in the stakeholder dialogue through letters and person-to-person invitations.

The dialogue will be conducted over the course of several sessions in which participants will gather in a neutral, public location to talk about the information described in the evidence brief. A neutral facilitator, who will ensure a respectful and equitable communication among participants, will moderate the stakeholder dialogue. This facilitator will be responsible for all participants having the same chance to express their views during the dialogue. The final products from the stakeholder dialogue will include a dialogue summary (ie, a distillation of the key themes and insights that emerged during the dialogue) and the formation of a complex csCBR program composed by different components or actions along with a clear description of their respective expected outcomes. It is anticipated that these actions will include individual, community, and societal targets.

The components of the csCBR program will be defined through a nonforced consensus achieved through a process agreed on by all participants at the beginning of the dialogue. Once the dialogue is completed, the csCBR program will be written, and the resulting document will be shared with all participants in order to assure its fidelity in relation to what was agreed during the dialogue. Agreements with powerful allies will be confirmed and clinched by signing letters of commitment. This strategy aims to favor the long-term sustainability of the csCBR program within each targeted community.

### Stage Three: Program Implementation

This stage will be completed over 6 months following a “quantitative dominant” approach [42]. The stage involves conducting a pilot test of the CBR program developed during stage two, and the implementation of an improved CBR program in the two communities of interest. The pilot test will help in identifying barriers and facilitators for the program’s implementation, allowing corresponding program adjustments. All partnership members will contribute to the design, execution, and interpretation of the results of this stage.

We will choose 1 strategic site at each community (ie, 1 commissariat in Chankom and 1 health center of the UHC-Nuevo León) to implement the csCBR program designed during stage two of this protocol. An anthropologist will assess the operational aspects of the csCBR program using nonparticipant observations, informal interviews, in-depth interviews, and focus groups. This qualitative information will be used to design two questionnaires to evaluate the presence of facilitators and barriers for the implementation of each of the components of the csCBR program in the community. There will be one questionnaire designed for users of the program and another one for personnel involved in the program’s execution. Trained interviewers will apply the questionnaires to all participants of the pilot test through home visits, visits at jobsites, or telephone calls.

Qualitative data will be analyzed and interpreted by the anthropologist and some members of the partnership using content and thematic analysis techniques. This analysis then will be presented to all partners to decide the content of the questionnaires. We will use descriptive statistics to rank the frequency of facilitators and barriers observed during the pilot test. The partnership will use this information to make decisions about relevant changes to the original csCBR program and to elucidate implementation strategies aiming to improve its successful implementation in the community. Once changes have been made, we will proceed to implement the updated csCBR program in both communities.

### Stage Four: Program Evaluation

This stage will last for 18 months following a “pure mixed methods” approach [42] implying the simultaneous execution of quantitative and qualitative methods, each one producing results that will converge in a complete explanation of the researched phenomenon [73]. The objectives of this stage are: (1) to understand which components of the csCBR program are more effective, and what are their mechanisms of action; and (2) to evaluate the impact of the csCBR program on the functioning and quality of life (QoL) of the people living with musculoskeletal diseases in Chankom and the community-UHC-Nuevo León. This stage will allow us to get a complete explanation and understanding about the impact and mechanisms of action of the csCBR program developed.

Quantitative methods will consist of a longitudinal, prospective, and comparative pre/post intervention observational design. Qualitative data will be gathered through ethnographic fieldwork to understand the dynamics and mechanisms of action of each of the csCBR program components. The ethnographic work will also inform quantitative findings about the impact of the program on functioning and QoL.

The quantitative sampling strategies will vary between our two target communities. In Chankom, we will include all the people enrolled in the CBR program together with a sample of people with equivalent ethnic, cultural, and socioeconomic characteristics, who live outside Chankom and have not been exposed to the program (control population). In the community-UHC-Nuevo León, we will assemble a random probabilistic sample of people with musculoskeletal diseases who are involved in the CBR program, and an equal sample of people with osteoarthritis living in a community with similar socioeconomic and cultural characteristics as the community-UHC-Nuevo León, but that has not been in contact with the program (control population). Quantitative results of stage one will provide us with the information needed to calculate appropriate sample sizes. The ethnographic work will require purposeful sampling of people who participated in activities that were implemented in the CBR program for at least 3 months, in both target communities. This will assure that sufficient experience with the program’s processes and activities has been accumulated.

For the quantitative part, we will take baseline measurements, prior to the implementation of the program, and follow-up measurements every 6 months (4 measurements in total until 18 months) in both the target and control populations. Subjects of the control populations will be identified using the COPCORD screening methodology described in stage one of the project. We will measure: (1) 3 different tenses of physical function [53]; (2) QoL; and (3) outcomes related to each component of the csCBR program, whatever these may be.

As already mentioned, we anticipate that the csCBR program will include interventions at different levels, from the personal to the institutional level. In consequence, outcomes will be defined and measured according to the theoretical understanding of each level.

Hypothetical functioning will be measured through the WHO Disability Assessment Schedule 2 (WHODAS 2.0). The WHODAS 2.0 is a generic health-related disability assessment with excellent psychometric properties and was created through an extensive multicultural effort [74]. Experimental functioning will be evaluated using the 6MWT and the FDT. Both tests have shown excellent psychometric properties in musculoskeletal disease populations [57,59]. Enacted functioning will be measured subjectively through the Patient-Specific Functional Scale, which has shown excellent validity and reliability properties when applied in musculoskeletal-related pain populations [75], and semiobjectively using self-report, nonparticipant observations, and videos. QoL will be assessed through the WHO QoL Instrument. This instrument was developed through a multicultural collaboration and has been used with different populations, including older adults, showing excellent reliability and validity properties [76]. All questionnaires will be translated and culturally adapted to the Mayan language.

The ethnographic fieldwork will be conducted by a medical anthropologist and will include participant and nonparticipant observations, in-depth interviews, and focus groups. These qualitative methodologies will be conducted to understand the mechanisms of action of the different components of the program, along with their respective positive and negative aspects. In addition, the fieldwork data will help us in identifying relevant effects of the csCBR program, which can be measured quantitatively.

We will include descriptive and inferential statistic techniques to analyze the quantitative data. Inferential techniques will include multilevel modeling to explore effect modifiers on the outcomes of interest at different levels (eg, municipality, commissariat, or household levels), including between-group comparisons among target and control populations. We will use the statistical software STATA version 12. Ethnographic data will be analyzed following an analytic-interpretative-reflexive strategy from a medical anthropology perspective. These analyses will be further enriched by discussions with the partnership. All analytic and methodological decisions will be carefully registered in an audit trail. The results of this stage four will support decision-making processes within the partnership, allowing planning and conducting of a new situational analysis, thus completing the cyclical nature of the project (see Figure 1). The cyclical nature of this project implies that the csCBR program's components will be constantly refined, and the outcomes expected by their implementation will be obtained after the execution of several cycles.

## Results

The complexity of this project poses challenges for obtaining funding. Funding agencies in the developing world lack awareness of the need for this type of project and knowledge about the use of mixed methodologies. As such, we used different strategies for communicating the methods of the project to different audiences. In addition, we have applied for funding at diverse agencies, asking separate support for conducting the different parts of the project.

So far, we obtained resources from a GLADERPO study, founded by a Mexican federal agency, and completed stage one of the project at Chankom.  We are currently receiving funding from an international agency to complete stage two at this same location. We expect that the project at Chankom will be concluded by December of 2017. On the other hand, we just started the execution of stage one at the community-UHC-Nuevo León with funding from a Mexican federal agency. We expect to conclude the project at this site by September of 2018.

## Discussion

### An Alternative Approach

This project represents an alternative approach for developing csCBR programs for low-income communities. This alternative considers both the research and the practice involved for the creation and execution of this type of program, and follows a participatory research approach. The main theoretical assumptions that give foundation to this project are: (1) a partnership between the community and academia is ideal, because they have different, noncompetitive, but yet complementary agendas (communities are more interested in their social development and well-being, while academia is more interested in producing and disseminating knowledge); (2) it is possible to construct new knowledge from the fusion of horizons between the community and academia; (3) reaching agreement through communicative practices will result in actions that promote social change; and (4) it is possible to build, understand, and evaluate complex multilevel interventions through the application of quantitative and qualitative methods.

The primary motivation behind this project is a need for interventions directed to reducing musculoskeletal-related disability identified by health professionals and academics. This need was informed by diverse experiences of professionals and researchers interacting with disabled people in low-income communities. Therefore, this project is the result of a genuine real life concern about the lack of social justice present in the lives of people living with musculoskeletal diseases in low socioeconomic geographic locations.

Historically, the development of CBR programs within developed and developing countries have presented some issues. These issues include the “one size fits all” strategy that is used to build such programs without considering the gap between what is needed and what is available within a community [77]. This is linked to the fact that many CBR programs have tried to import the model of “hospital rehabilitation care” directly to the community [31], resulting in “disempowering” practices [78] that aim to empower individuals without addressing “social inequalities” [79].

Our approach to csCBR program development acknowledges such problems and tries to address them through the application of a mixed method program that is “cognizant, appreciative, and inclusive of local sociopolitical realities, resources, and needs” [42]. This means that each community has to be considered as a unique entity and a general approach to build csCBR programs should incorporate efforts for adaptation to local contexts. In addition, we are proposing a grass-roots approach through the CBPR strategy. Instead of empowering individuals, this approach will aim to redistribute power, equalizing it between members of the community and academia. We believe that this strategy will counteract the inequality produced by the “charity model” [25,26] adopted by the welfare state of Mexico. In other words, instead of using the CBR program as a “band-aid” approach for solving immediate community problems [25], we are trying to promote the creation of democratic actions towards social change.

Projects of this nature will always be at risk of generating power imbalances between the members of the partnership and between the partnership and the powerful allies. This is why we are incorporating a real transdisciplinary collaboration, which involves the community and representatives from the health and social sciences. The work performed by the social scientist(s) within each stage of the project will help to disclose power imbalances, induce reflection about them, and remediate power differentials over time. This will also help to give a sense of ownership of the CBR program to all participants within the partnership and to make the collaboration with powerful allies more efficient.

Another substantial issue, registered during the development of CBR programs, is the lack of proper research and evaluation of the effects that these programs have on the disablement process within communities [41]. It is evident that evaluating these types of complex interventions is conceptually challenging. Using the traditional randomized controlled trial (RCT) approach is not feasible because of its lack of in-depth examination of the social, cultural, and organizational factors that could influence outcomes [80]. In addition, it is almost impossible to use randomization procedures within the real life situations in which CBR programs are implemented [80]. Finally, the information gathered through an RCT does not allow the capturing of the interactions between the individuals and their social and physical environments [80].

Our approach to the problem of evaluating CBR programs is to incorporate mixed methods research, in which both qualitative and quantitative methods are executed either in sequence or in parallel [42]. This implies the execution of quantitative and qualitative techniques, each one producing results that either will inform one another or will converge on a complete explanation of what is researched [73].

We opted for an ethnographic approach, due to our need for understanding the knowledge, values, and emotions towards musculoskeletal disability of people living in low-income communities within their natural settings. On the other hand, we are taking a quantitative prospective and observational approach, which will allow the use of powerful statistic tools such as multilevel analysis [81]. In addition, we are considering executing some cost-effectiveness analyses to inform the policy arena. However, at this point we would rather wait until the partnerships are well established to make decisions on how to proceed about cost analyses in the project.

### Differences Between Communities

There are important differences between the community-UHC-Nuevo León and the community of Chankom. These differences have methodological and organizational implications. Nuevo León’s community is 100% urban, while the Mayan community of Chankom is completely rural. This situation influences the type and consequences of existing disabling situations within these communities. The community-UHC-Nuevo León is immersed in one of the most violent Mexican States, while Chankom is situated in the least violent state of Mexico, Yucatan. This could have many repercussions on the feasibility of conducting real ethnographic work in Nuevo León because of the need for the researcher to live there for a period of time. A solution could be to locate and involve local social scientists in that area. In addition, there are important differences between communities in relation to size and spoken language. Chankom is a small indigenous community with little more than 4000 individuals who mostly speak Mayan; meanwhile, community-UHC-Nuevo León has more than 140,000 Spanish-speaking individuals. This will require constant translation efforts and the use of a significant amount of human resources. Differences between our target communities will allow us to compare between sites, advancing our understanding of the methodology required to conduct this type of project.

Another important difference between the sites involved in this project relates to the status of their local health structures and community organization development. The community-UHC-Nuevo León has a strong local primary health care system embedded in a well organized community. Whereas, there is no local health care system in Chankom and the community is poorly organized to confront their health problems. Consequently, in the community-UHC-Nuevo León we will include and share power with the community through collaboration with local health providers and community leaders since the first stage (situational analysis) of the project, which is the traditional CBPR approach. However, in Chankom we are taking a modified CBPR approach in the sense that the situational analysis will be conducted as a project driven by people from outside the community. This strategy aims to use the initial research efforts and results to motivate community organization, which will facilitate the establishment of an authentic partnership for the conduction of the next stages of the project. Chankom’s situation exemplifies the difficulties encountered by trying to apply an approach developed in more organized communities to a community where organization for solving health issues is nonexistent, as are the majority of poor rural communities in Mexico.

### Conclusions

In conclusion, this project is intended to move forward the methodology for the development of csCBR programs in low-income communities. These programs will contribute to community development of these Mexican socially marginalized areas and will cover the need to receive adequate health care for people living with musculoskeletal diseases at these locations.

## Abbreviations

6MWT: 6-minute walk test
CBR: community-based rehabilitation
CBPR: community-based participatory research
community-UHC-Nuevo León: community served by the University Health Center of Nuevo León, Monterrey, México
COPCORD: Community Oriented Program for the Control of Rheumatic Diseases
csCBR: culturally sensitive community based rehabilitation
FDT: functional dexterity test
GLADERPO: Grupo Latinoamericano para el Estudio de Enfermedades Reumaticas en Poblaciones de Origen Grupo (Latin American Group for the Study of Rheumatic Conditions in Indigenous People)
HAQ-DI: Health Assessment Questionnaire Disability Index
QoL: quality of life
RCT: randomized controlled trial
UHC-Nuevo León: University Health Center of Nuevo León, Monterrey, México
WHO: World Health Organization
WHODAS 2.0: WHO Disability Assessment Schedule 2
